# Type 2 diabetes mellitus worsens neurological injury following cardiac arrest: an animal experimental study

**DOI:** 10.1186/s40635-018-0193-2

**Published:** 2018-08-07

**Authors:** Lauge Vammen, Søren Rahbek, Niels Secher, Jonas Agerlund Povlsen, Niels Jessen, Bo Løfgren, Asger Granfeldt

**Affiliations:** 1 Department of Intensive Care Medicine, Aarhus University Hospital, Aarhus, Denmark; 2 Research Center for Emergency Medicine, Aarhus University Hospital, Aarhus, Denmark; 3 Department of Cardiology, Aarhus University Hospital, Aarhus, Denmark; 4 Department of Clinical Pharmacology, Aarhus University, Aarhus, Denmark; 5 Department of Internal Medicine, Regional Hospital of Randers, Randers, Denmark

**Keywords:** Cardiac arrest, Resuscitation, Animal model, Type 2 diabetes mellitus, Neurological injury, Echocardiography

## Abstract

**Background:**

Cardiac arrest carries a poor prognosis. The typical cardiac arrest patient is comorbid, and studies have shown that diabetes mellitus is an independent risk factor for increased mortality after cardiac arrest. Despite this, animal studies lack to investigate cardiac arrest in the setting of diabetes mellitus.

We hypothesize that type 2 diabetes mellitus in a rat model of cardiac arrest is associated with increased organ dysfunction when compared with non-diabetic rats.

**Methods:**

Zucker diabetic fatty (ZDF) rats (*n* = 13), non-diabetic Zucker lean control (ZLC) rats (*n* = 15), and non-diabetic Sprague Dawley (SprD) rats (*n* = 8), underwent asphyxia-induced cardiac arrest. Animals were resuscitated and monitored for 180 min after return of spontaneous circulation (ROSC).

Blood levels of neuron-specific enolase were measured to assess neurological injury. Cardiac function was evaluated by echocardiography.

**Results:**

No differences in cardiac output or neuron-specific enolase existed between the groups at baseline. Median levels of neuron-specific enolase 180 min after ROSC was 10.8 μg/L (Q_25_;Q_75_—7.6;11.3) in the ZDF group, which was significantly higher compared to the ZLC group at 2.0 μg/L (Q_25_;Q_75_—1.7;2.3, *p* < 0.05) and the SprD group at 2.8 μg/L (Q_25_;Q_75_—2.3;3.4, *p* < 0.05). At 180 min after ROSC, cardiac output was 129 mL/min/kg (SD 45) in the ZDF group, which was not different from 106 mL/min/kg (SD 31) in the ZLC group or 123 mL/min/kg (SD 26, *p* = 0.72) in the SprD group.

**Conclusions:**

In a cardiac arrest model, neuronal injury is increased in type 2 diabetes mellitus animals compared with non-diabetic controls. Although this study lacks to uncover the specific mechanisms causing increased neuronal injury, the establishment of a cardiac arrest model of type 2 diabetes mellitus lays the important foundation for further experimental investigations within this field.

## Background

Cardiac arrest carries a poor prognosis. Organ injury following cardiac arrest is initiated by the ischemic insult and is paradoxically worsened by concomitant reperfusion, also known as ischemia-reperfusion (IR)-injury. In addition to systemic IR-injury, the post-cardiac arrest syndrome consists of myocardial dysfunction, brain injury, and the persisting precipitating pathology [1].

A precipitating pathology that may co-exist with cardiac arrest is type 2 diabetes mellitus (T2DM). Recent large clinical trials and retrospective studies have shown that the prevalence of diabetes mellitus is more frequent in cardiac arrest patients (13–28%) [2–5] than in the background population (approx. 6%) [6]. Following out-of-hospital cardiac arrest, patients with diabetes mellitus show reduced neurological recovery compared to non-diabetic patients [4, 7]. Importantly, several retrospective studies have demonstrated that diabetes mellitus independent of other risk factors is a strong predictor of poor outcome after cardiac arrest [8–11]. The exact mechanism behind this association is unknown. It may relate to augmented IR-injury which has been demonstrated in animal models of diabetes mellitus in organ-specific experiments [12–15]. However, in the setting of cardiac arrest, most experimental animal studies are conducted on healthy young animals [16], limiting our knowledge regarding the pathophysiology of the post-cardiac arrest syndrome and the impact of co-morbidities.

## Methods

### Study aim

The objectives of this study were to (1) establish a cardiac arrest animal model of T2DM and (2) to investigate the effect of T2DM on organ dysfunction following cardiac arrest. We hypothesize that T2DM is associated with increased neurological injury and cardiac dysfunction.

### Experimental animals and housing

Three different groups of animals were used in the current study: (1) 16–18-week-old male Zucker diabetic fatty (ZDF) rats (fa/fa), (2) 16–18-week-old male Zucker lean control (ZLC) rats (fa/+) (Charles River Laboratories, Kingston, NY, USA), and age-matched male Sprague Dawley rats (SprD) (Janvier Labs, Le Genest-Saint-Isle, France). Both ZDF and ZLC rats originate from the same inbred strain of rats and shares an identical genetic background apart from a genetic mutation in the leptin receptor and defect in pancreatic β-cell transcription in the ZDF rats. [17]*.*

During the pilot studies, a low rate of return of spontaneous circulation (ROSC) was observed in the ZLC group compared to ZDF animals. The SprD group was included to ensure a sufficiently powered healthy control group in case of a continued low ROSC rate in the ZLC group during the experiments. All animals were housed in pairs at standard room temperature, with a 12-h light/12-h dark cycle with free access to food and water. ZDF and ZLC rats were fed high-fat diet (Purina #5008, LabDiet, St. Louis, MO, USA) as recommended by the supplier, while SprD rats were fed standard rodent diet (Altromin 1324, Lage, Germany).

### Study design

**Fig. 1:**
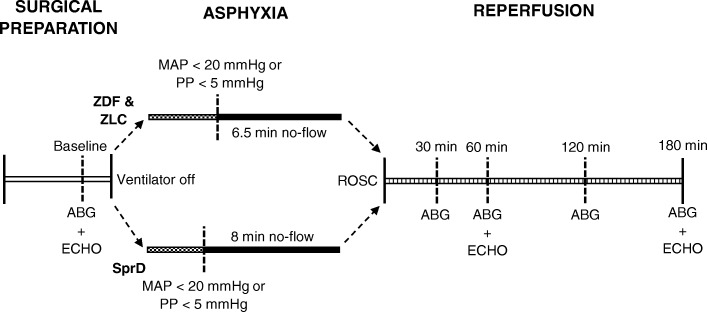
Study design. Surgical preparation and monitoring during reperfusion were similar between all the three groups. During asphyxia, ZDF and ZLC groups underwent 6.5 min no-flow (top tier) and the SprD group underwent 8 min no-flow (lower tier), to ensure similar total asphyxia time. MAP: mean arterial pressure, PP: pulse pressure, ROSC: return of spontaneous circulation, ABG: arterial blood gas, ECHO: echocardiography, ZDF: Zucker diabetic fatty group, ZLC: Zucker lean control group, SprD: Sprague Dawley group

Animals underwent asphyxia-induced cardiac arrest in a randomized order with regard to the group by drawing pieces of paper from an envelope. Phenotypical differences made it impossible to blind the investigator to the experimental groups during experiments. The ischemic insult during asphyxial cardiac arrest consists of time to cardiac arrest and no-flow time. Based on the pilot experiments, the no-flow time in the ZDF and ZLC rats was set to 6.5 min. Similarly, we experienced that time to cardiac arrest was of shorter duration in the SprD group compared with the two other groups. Consequently, the no-flow time in this group was extended to 8 min to have comparable total asphyxia times between SprD and ZDF groups (see Fig. 1).

### Diabetes diagnosis

Animals were fasted for 5 h the day prior to cardiac arrest. To confirm diabetes, three tail capillary blood samples were collected, and blood glucose levels were measured (OneTouch UltraEasy Glucometer, Roche Diagnostics, Copenhagen, Denmark). Diabetes was defined as an average fasting blood glucose level > 7.0 mmol/L.

### Animal and surgical preparation

On the day of surgery, anesthesia was induced by 8% sevoflurane (Sevorane, AbbVie, Copenhagen, Denmark) in a chamber and maintained by 4% sevoflurane after intubation. The ventilator (MRI-1 ventilator, CWE Inc., PA, USA) was set to a tidal volume of 9 mL/kg with 1 cm positive end-expiratory pressure and fraction of inspired oxygen (FiO_2_) at 30%. Respiratory rate was adjusted to maintain arterial partial pressure of CO_2_ (PaCO_2_) between 4.7 and 6.0 kPa. The left femoral artery and vein were catheterized using polyethylene tubing (PE 90 and PE 50, Intramedic™, Copenhagen, Denmark), while mikro-tip catheters (Millar Instruments, TX, USA) were introduced into the right common carotid artery to the aortic root and into the external jugular vein to the right atrium. To prevent dehydration and clotting of catheters, 1 mL/kg of heparinized saline (2.5 IU/mL; Heparin, LEO Pharma, Malmö, Sweden) was administered every 30 min before cardiac arrest.

### Experimental protocol

To inhibit spontaneous respiration during induction of cardiac arrest, 2.4 mg/kg rocuronium (Hameln Pharma plus, Hameln, Germany) was administered intravenously as a bolus before cardiac arrest. Immediately after the administration of rocuronium, both sevoflurane and positive end-expiratory pressure were discontinued. After a 30-s washout period, the ventilator was turned off and the inspiratory tube clamped. Cardiac arrest was defined as a mean arterial pressure (MAP) below 20 mmHg and/or a pulse pressure lower than 5 mmHg.

Resuscitation was initiated with adrenaline (0.01 mg/kg), mechanical chest compressions by a custom-made thumper (set to 200 bpm and a depth of one third of the height of the thorax), and ventilation with a respiratory rate of 110 min^−1^ and FiO_2_ of 100%. Resuscitation was continued for up to 5 cycles of 2 min with heart rhythm analysis between cycles, followed by adrenaline administration. If a shockable rhythm occurred, up to three stacked shocks (4 J) were delivered through electrocardiogram electrodes placed on the front and back of the thorax. If a shockable rhythm was present at subsequent rhythm analyses, another shock was delivered. Chest compressions were resumed if conversion to a perfusing rhythm failed and/or a MAP below 40 mmHg persisted.

Successful resuscitation was defined as MAP above 40 mmHg. Immediately after ROSC, sevoflurane was reinitiated at 2% for the remainder of the experiment. If the MAP dropped below 40 mmHg following ROSC during the first 30 min after ROSC, adrenaline support (0.0015 mg/kg) was given. All animals were monitored for 180 min after ROSC. During monitoring, FiO_2_ was adjusted to maintain arterial oxygen saturation above 94%. After resuscitation, to prevent dehydration, ZLC and SprD rats received 1 mL/kg of heparinized saline every 2 h, while the ZDF rats received the same dose every hour due to glycosuria and increased urine output. Seizures during the reperfusion period were defined as visible generalized tonic-clonic muscle contractions.

### Monitoring

All animals were monitored with hind limb saturation, one-lead electrocardiogram, blood pressure, and heart rate during the entire experiment. The temperature was measured by a rectal probe and was kept at 37 ± 0.5 °C during the entire experimental protocol. Blood gasses (45 μL per sample) were collected from the femoral arterial catheter and analyzed (ABL 90 Flex Plus, Radiometer, Copenhagen, Denmark) at baseline, 30, 60, 120, and 180 min after ROSC. The coronary perfusion pressure (CPP) was calculated during resuscitation, as the difference between end-diastolic aortic and right atrial pressure measured by the mikro-tip catheters, as the average of five compressions, 20 s after the start of cardio-pulmonary resuscitation.

### Biochemistry

Blood samples were drawn at baseline and at the end of the experiment; serum samples (1 mL) to measure neuron-specific enolase (NSE) and EDTA samples (0.75 mL) to measure troponin T (TnT), inflammatory cytokines (IL-1β, IL-6, and IL-10), and adhesion molecules (soluble L-selectin [sL-selectin] and soluble ICAM-1 [sICAM-1]). The serum samples clotted for 20–30 min at room temperature before they were centrifuged at 4 °C and 1850*g* for 9 min, while the EDTA samples were centrifuged immediately at 4 °C and 1500*g* for 20 min. The supernatant was kept at − 80 °C before later analysis. NSE and TnT were analyzed on Cobas 6000 e601 (Roche Diagnostics, Copenhagen, Denmark). Inflammatory cytokines and adhesion molecules were analyzed using a multiplex assay (R&D Systems Europe, Abingdon, UK). The assay was analyzed on a Luminex^®^100 using the BioPlex Software Manager 5.0 (Bio-Rad, Hercules, CA, USA). For all tests, a single analysis was made, and the investigator was blinded to both experimental group and examination time. Detection limits and intra-assay variation coefficients were as follows: NSE—0.05 μg/L, 1.2%; TnT—5 ng/L, 1.7%; IL-1β—32.3 pg/mL, 6.9%; IL-6—384.9 pg/mL, 7.9%; IL-10—22.9 pg/mL, 5.8%; sL-selectin—206 pg/mL, 7.4%; and sICAM-1—33.7 pg/mL, 6.5%.

### Echocardiography

Echocardiographic examinations were performed at baseline, 60 and 180 min after ROSC (see Fig. 1). A stabilization period was allowed for baseline recordings between surgical preparations and cardiac arrest. Transthoracic 2D echocardiography of the left ventricle was performed with a GE Healthcare Vivid S6 ultrasound system using an 11-MHz probe (12S-RS, GE Healthcare, Copenhagen, Denmark). All image analyses were performed after the experiments (EchoPac, GE Healthcare, Copenhagen, Denmark), randomized and blinded with regard to both experimental group and examination time. In the apical five-chamber view, pulsed waved Doppler was applied to measure the aortic outflow, and from this, the velocity time integral and heart rate were measured for three consecutive cardiac cycles. The left ventricular outflow tract (LVOT) diameter was measured in the parasternal long axis view, and from this, the LVOT area was calculated. Cardiac output (mL/min) was manually calculated and standardized to individual animal weight using the following formula: (LVOT-area in cm^*2*^ × LVOT-VTI mean in cm × heart frequency per min)/animal weight in kg. In the parasternal short axis view (below mitral leaflets with visible papillary muscles), M-mode analysis was applied to evaluate the left ventricle during both diastole and systole for three consecutive cardiac cycles. The following dimensions were measured: interventricular septum thickness during both diastole (IVSD) and systole (IVSS), left ventricular inner diameter (LVIDD and LVIDS), and left ventricular posterior wall thickness (LVPWD and LVPWS). Left ventricular function was furthermore evaluated by fractional shortening: ((LVIDD − LVIDS)/LVIDD) × 100%. To evaluate diastolic function, pulsed waved Doppler was applied in the apical four-chamber view to measure the mitral valve inflow, with subsequent analysis of E/A ratio as an average over five cardiac cycles.

To determine intraobserver variability, 20 randomly selected images for each analysis were re-analyzed. The mean difference and 95% confidence limits were 3.87 mL/min/kg (− 8.08; 15.83) for cardiac output from velocity time integral, 0.15% (− 5.96; 5.67) for fractional shortening, and 0.01 (− 0.19; 0.21) for E/A ratio.

### Statistical analysis

Normally distributed data are presented as mean and standard deviation (SD), and non-normally distributed data are presented as median with associated 25% and 75% quartiles (Q_25_ and Q_75_). Normality was determined by histograms and Q-Q plots. It was a priori determined that all comparisons are made between ZDF and ZLC rats or ZDF and SprD rats. No comparison between the control groups was performed. Due to the low number of animals with echocardiographic parameters in the ZLC group, echocardiographic parameters were only compared between the ZDF and SprD groups. Non-normally distributed data were transformed on a logarithmic scale to assume normality if possible, but all data in figures and tables are presented on the original scale. Repeated measurements were analyzed by repeated measurements analysis of variance (ANOVA) to detect difference over time between the groups. Baseline and end-of-experiment between-group differences were analyzed using Student’s *t* test or Mann-Whitney *U* test as appropriate. Continuous variables were analyzed using Student’s *t* test or Mann-Whitney *U* test. Dichotomous outcomes were compared using Fischer’s exact test.

Analyses and figures were performed using Stata 12.1 (StataCorp, College Station, TX, USA) and Prism 6.07 (GraphPad, La Jolla, CA, USA). A *p* value below 0.05 was considered significant.

## Results

A total of 40 animals were included in this study. Four animals were excluded: two ZDF rats due to technical difficulties prior to arrest, one SprD rat due to breach of resuscitation protocol, and one SprD rat developed severe hypoglycemia after cardiac arrest. The remaining 36 animals were distributed as follows: (1) ZDF group (*n* = 13), (2) ZLC group (*n* = 15) rats, and (3) SprD rats (*n* = 8). The mean weight was 422 g (SD 46) in ZDF group, 353 g (SD 23, *p* < 0.05) in the ZLC group, and 460 g (SD 29, *p* = 0.05) in the SprD group.

### Fasting blood glucose levels

All ZDF rats developed diabetes (fasting blood glucose > 7 mmol/L). Median fasting glucose levels were significantly higher in the ZDF group at 19.9 mmol/L (Q_25_;Q_75_—19.4;21.2) when compared with the ZLC group at 5.0 mmol/L (Q_25_;Q_75_—4.7;5.2, *p* < 0.05) and SprD group at 5.1 mmol/L (Q_25_;Q_75_—5.0;5.3, *p* < 0.05).

### Cardiac arrest and resuscitation

Data on cardiac arrest and resuscitation are presented in Table 1. Time to cardiac arrest differed significantly between the groups with the longest duration in the ZDF group when compared with both the ZLC group (*p* < 0.05) and the SprD group (*p* < 0.05). Because of a longer no-flow time in the SprD group, no difference in the total asphyxia time existed between the ZDF and SprD groups (*p* = 0.60); it was, however, significantly longer in the ZDF group when compared with the ZLC group (*p* < 0.05).

**Table 1:** Cardiac arrest and resuscitation

Group	Time to cardiac arrest (s)	Total asphyxia time (s)	Time to ROSC (s)	ROSC rate^‡^	CPP (mmHg)^§^
ZDF	249 (24)	639 (24)	42 (38;46)	11/13	33 (14)
ZLC	202 (18)*	592 (18)*	75 (60;224)	6/15*	18 (13)*
SprD	154 (20)^†^	634 (20)	52 (39;57)	8/8	20 (11)^†^

Data are presented as mean (SD) or median (Q_25_;Q_75_). *p < 0.05 ZDF vs. ZLC. ^†^*p* < 0.05 ZDF vs. SprD. ^‡^Animals achieved ROSC/total number of animals in group. ^§^CPP 20 s after the start of resuscitation. *S* seconds, *ROSC* return of spontaneous circulation, *CPP* coronary perfusion pressure, *ZDF* Zucker diabetic fatty group, *ZLC* Zucker lean control group, *SprD* Sprague Dawley group

The rate of ROSC varied significantly between ZDF (11/13) and ZLC (6/15) animals (*p* < 0.05), but not between ZDF and SprD animals (8/8, *p* = 0.51). The CPP 20 s after the start of cardio-pulmonary resuscitation was significantly higher in the ZDF group compared with both control groups (ZLC *p* < 0.05 and SprD *p* < 0.05). During resuscitation, one ZDF rat and two ZLC rats were defibrillated due to ventricular fibrillation, of which one from the ZLC group was successfully converted to sinus rhythm and survived throughout the experimental protocol. Three animals in total (all ZDF rats) received adrenaline support during the initial 30 min of reperfusion. This was not statistically significant when compared to control groups (ZLC *p* = 0.52 or SprD *p* = 0.23).

### Physiology

Physiologic parameters are presented in Table 2*.* The MAP was lower in the SprD group (*p* < 0.05) at baseline compared with the ZDF group. Within the first 120 min after ROSC, MAP was higher in the ZDF group compared with both control groups, but no significant difference was observed between the groups after 180 min. At the end of the experiment, pH and base excess (BE) were significantly lower in the ZDF group compared with the ZLC group (*p* < 0.05 for both variables) and the SprD group (*p* < 0.05 for both variables). Higher potassium levels were also seen in the ZDF group compared with the ZLC group (*p* < 0.05) and SprD group (*p* < 0.05). After ROSC, lactate levels were elevated in the ZDF group. Furthermore, at the end of the experiment, lactate in the ZDF group was significantly higher than in the ZLC group (*p* < 0.05) and the SprD group (*p* < 0.05). Blood glucose levels were significantly higher in the ZDF group when compared with both the ZLC and SprD groups (*p* < 0.05 at baseline and end of the experiment for both groups). PaO_*2*_/FiO_*2*_ levels were significantly lower in the ZDF group at baseline when compared to SprD rats with no difference between ZLC and ZDF groups. PaO_*2*_/FiO_*2*_ increased over time in the ZDF group and decreased in ZLC and SprD groups with significantly higher levels in the ZDF group at the end of the experiment.

**Table 2:** Mean arterial pressure, heart rate, temperature, and blood gas analysis

	GROUP	Baseline	30 min ROSC	60 min ROSC	120 min ROSC	180 min ROSC	*p*-value^‡^
MAP, mmHg	ZDF	103 (15)	107 (41)	95 (32)	97 (27)	53 (20)	
	ZLC	98 (11)	97 (41)	47 (7)	52 (7)	55 (14)	< 0.05
	SprD	77 (10)^†^	74 (25)	49 (4)	54 (14)	58 (12)	< 0.05
Temp, °C	ZDF	37.1 (0.2)	36.9 (0.2)	36.6 (0.2)	37.2 (0.4)	37.0 (0.3)	
	ZLC	37.0 (0.2)	36.9 (0.2)	37.0 (0.3)	37.0 (0.2)	37.1 (0.3)	< 0.05
	SprD	37.0 (0.1)	36.9 (0.1)	36.9 (0.2)	37.1 (0.1)	37.1 (0.2)	0.07
pH	ZDF	7.47 (0.05)	7.30 (0.09)	7.32 (0.08)	7.19 (0.07)	7.11 (0.10)	
	ZLC	7.46 (0.05)	7.31 (0.06)	7.32 (0.05)	7.32 (0.03)	7.32 (0.03)*	< 0.05
	SprD	7.49 (0.02)	7.34 (0.06)	7.37 (0.05)	7.39 (0.04)	7.38 (0.05)^†^	< 0.05
BE, mmol/L	ZDF	5.0 (3.7)	− 7.9 (3.4)	− 6.4 (3.1)	− 12.9 (4.0)	− 17.5 (5.1)	
	ZLC	4.0 (3.4)	− 5.4 (3.3)	− 4.5 (3.5)	− 3.4 (3.6)	− 4.4 (2.9)*	< 0.05
	SprD	5.9 (1.6)	− 3.4 (2.3)	− 1.0 (1.8)	− 0.4 (1.7)	− 1.3 (3.2)^†^	< 0.05
PaCO2, kPa	ZDF	5.3 (0.6)	5.0 (1.6)	5.1 (0.7)	5.2 (0.6)	4.9 (0.9)	
	ZLC	5.2 (0.3)	5.6 (1.2)	5.6 (1.0)	5.9 (0.6)	5.6 (0.4)	0.64
	SprD	5.1 (0.1)	5.5 (0.7)	5.7 (0.7)	5.5 (0.6)	5.3 (0.2)	0.47
PaO2/FiO2 ratio	ZDF	450 (518)	450 (270)	398 (255)	570 (323)	636 (368)	
	ZLC	518 (105)	270 (120)	255 (75)	323 (143)	368 (128)*	< 0.05
	SprD	563 (68)^†^	315 (143)	330 (120)	368 (83)	435 (150)^†^	< 0.05
Glucose, mmol/L	ZDF	25.9 (6.2)	39.5 (8.0)	39.5 (8.1)	41.9 (5.9)	43.9 (4.3)	
	ZLC	11.9 (2.2)*	12.8 (2.7)	9.8 (3.6)	7.4 (1.5)	5.4 (1.2)*	< 0.05
	SprD	11.6 (0.8)^†^	11.1 (0.8)	8.2 (0.8)	7.7 (0.5)	6.8 (2.1)^†^	< 0.05
Lactate, mmol/L	ZDF	2.2 (1.1)	6.4 (2.4)	5.0 (1.7)	7.4 (3.3)	8.2 (3.1)	
	ZLC	1.2 (0.4)	4.1 (1.2)	3.1 (1.5)	2.6 (1.7)	2.6 (1.7)*	< 0.05
	SprD	1.2 (0.5)	3.9 (0.9)	2.7 (0.7)	2.1 (0.8)	1.8 (0.9)^†^	< 0.05
K^+^, mmol/L	ZDF	4.0 (0.3)	5.4 (0.7)	6.1 (1.6)	6.4 (0.9)	7.9 (1.5)	
	ZLC	4.1 (0.4)	3.9 (0.4)	4.9 (1.0)	5.6 (0.8)	5.6 (0.5)*	< 0.05
	SprD	4.7 (0.3)^†^	4.3 (0.3)	5.4 (0.4)	6.0 (0.4)	5.6 (0.9)^†^	< 0.05

All data are presented as mean (SD). *p < 0.05 ZDF vs. ZLC at baseline or 180 min after ROSC. ^†^*p* < 0.05 ZDF vs. SprD at baseline or 180 min after ROSC. ^‡^Time/group interaction (rep. meas. ANOVA), ZDF vs. ZLC and ZDF vs. SprD. *ROSC* return of spontaneous circulation, *MAP* mean arterial pressure, *BE* base excess, *PaCO2* CO_2_ in arterial blood, *PaO2* O_2_ in arterial blood, *FiO2* fraction of O*2* in inspiratory air, *K*^*+*^ potassium, *ZDF* Zucker diabetic fatty group, *ZLC* Zucker lean control group, *SprD* Sprague Dawley group

### Neurological injury

**Fig. 2:**
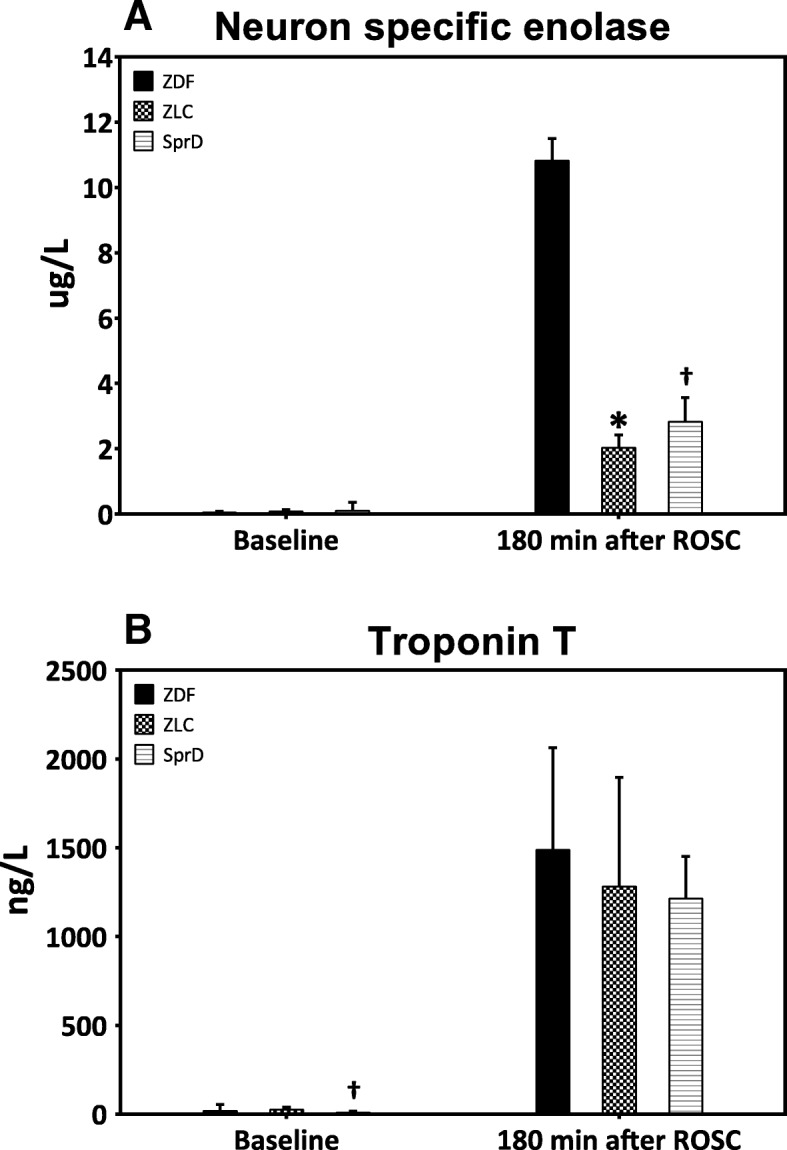
Neuron-specific enolase and troponin T. **a** Neuron-specific enolase values at baseline and 180 min after ROSC. Data are presented as median and associated quartiles (Q_25_ and Q_75_). Number of animals: ZDF (*n* = 10), ZLC (*n* = 6), and SprD (*n* = 8). **b** Troponin T values at baseline and 180 min after ROSC. Data are presented as mean and SD. Number of animals: ZDF (*n* = 11, one missing data point at 180 min after ROSC), ZLC (*n* = 6), and SprD (*n* = 8). For both analyses, only the animals achieving ROSC are included. *p < 0.05 ZDF vs. ZLC; ^†^*p* < 0.05 ZDF vs. SprD. ROSC: return of spontaneous circulation, ZDF: Zucker diabetic fatty group, ZLC: Zucker lean control group, SprD: Sprague Dawley group

The serum values of NSE from all the three groups are displayed in Fig. 2a. No difference was observed between groups at baseline. At the end of the study, median NSE was 10.8 μg/L (Q_25_;Q_75_—7.6;11.3) in the ZDF group which was significantly higher compared to the ZLC group at 2.0 μg/L (Q_25_;Q_75_—1.7;2.3, *p* < 0.05) and the SprD group at 2.8 μg/L (Q_25_;Q_75_—2.3;3.4, *p* < 0.05). Following cardiac arrest, all ZDF animals (11/11) had seizures, which was significantly more frequent compared with SprD animals (1/8, *p* < 0.05). No significant difference was observed with regard to the ZLC animals (4/6, *p* = 0.11).

### Cardiac function

At baseline, levels of TnT (Fig. 2b) were 28.1 ng/L (SD 25.7) in the ZDF group, 26.8 ng/L (SD13.3, *p* = 0.91) in the ZLC group, and significantly lower in the SprD group at 9.9 ng/L (SD 5.3, *p* < 0.05). No statistical significant difference was observed 180 min after ROSC between ZDF and ZLC groups (*p* = 0.48) or ZDF and SprD groups (*p* = 0.11).

**Fig. 3:**
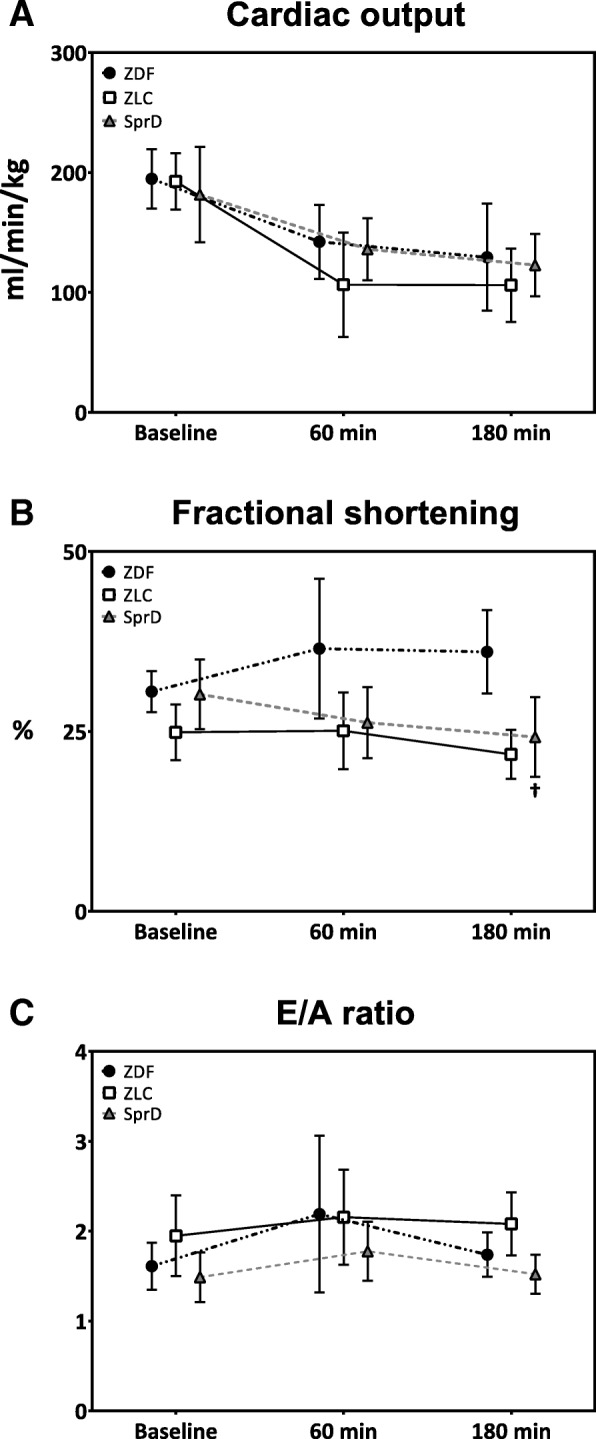
Echocardiographic parameters of cardiac function. Data are presented as mean and SD. **a** Cardiac output standardized to animal weight. Number of animals at examination times: ZDF (*n* = 13, 11, and 9), ZLC (*n* = 10, 5, and 2), and SprD (*n* = 8 at all examinations). **b** Fractional shortening; ZDF (*n* = 11, 11, and 10), ZLC (*n* = 14, 6, and 5), and SprD (*n* = 8 at all examinations). **c** E/A ratio; ZDF (*n* = 12, 10, and 8), ZLC (*n* = 14, 5, and 4), and SprD (*n* = 8,6, and 8). ^†^*p* < 0.05 ZDF vs. SprD. ZDF: Zucker diabetic fatty group, ZLC: Zucker lean control group, SprD: Sprague Dawley group

Cardiac output standardized to weight (Fig. 3a) was not statistically different between ZDF and SprD groups at baseline (*p* = 0.47) or at 180 min after ROSC (*p* = 0.72).

**Fig. 4:**
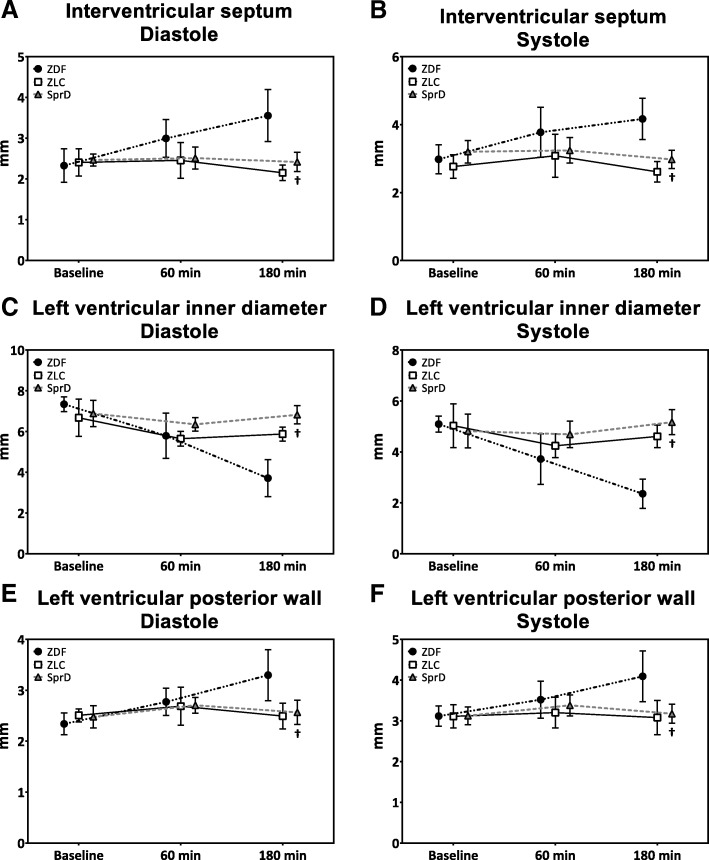
Left ventricular dimensions. Data are presented as mean and SD. **a** Interventricular septum in diastole. **b** Interventricular septum in systole. **c** Left ventricular inner diameter in diastole. **d** Left ventricular inner diameter in systole. **e** Left ventricular posterior wall in diastole. **f** Left ventricular posterior wall in systole. Number of animals at examination times: ZDF (*n* = 11, 11, and 10), ZLC (*n* = 14, 6, and 5), and SprD (*n* = 8 at all examinations). ^†^*p* < 0.05 ZDF vs. SprD. ZDF: Zucker diabetic fatty group, ZLC: Zucker lean control group, SprD: Sprague Dawley group

No difference was observed between ZDF and SprD groups in fractional shortening at baseline (Fig. 3b). At the end of the experiment, fractional shortening was 36% (SD 6) in ZDF group, which was significantly higher than 24% (SD 6, *p* < 0.05) in the SprD group. No differences in E/A ratio were observed between the groups throughout the experimental period (Fig. 3c). The left ventricular dimensions (Fig. 4a–f) were similar at baseline between ZDF and SprD groups. At 180 min after ROSC, however, the IVS and LVPW was statistically significantly thicker, and LVID statistically significantly smaller in the ZDF group compared with the SprD group. This was true during both diastole and systole.

### Inflammatory cytokines and adhesion molecules

**Fig. 5:**
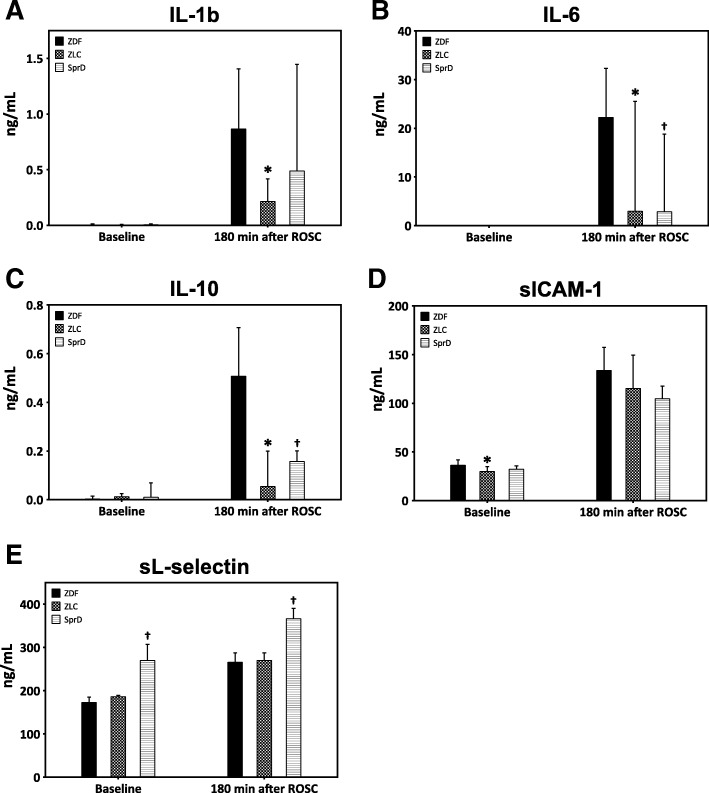
Cytokine and soluble endothelial adhesion molecules. Data are presented as median and associated quartiles (Q_25_ and Q_75_). **a** IL-1b. **b** IL-6. **c** IL-10. **d** Soluble ICAM-1. **e** Soluble L-selectin. Number of animals: ZDF (*n* = 11, one missing data point at 180 min after ROSC), ZLC (*n* = 6), and SprD (*n* = 8). For all analyses, only the animals achieving ROSC are included. *p < 0.05 ZDF vs. ZLC; ^†^*p* < 0.05 ZDF vs. SprD. ROSC: return of spontaneous circulation, ZDF: Zucker diabetic fatty group, ZLC: Zucker lean control group, SprD: Sprague Dawley group

Figure 5 shows the levels of cytokines and soluble endothelial adhesion molecules in the plasma. No difference in the cytokines was observed at baseline between the groups. All cytokine levels increased following ROSC. At the end of the experiment, the ZLC group had significantly lower levels of IL-1β, IL-6, and IL-10 compared with the ZDF group (*p* < 0.05 for all three cytokines), and the SprD group had significantly lower levels of IL-6 and IL-10 compared to the ZDF group (*p* < 0.05 for both cytokines). Both sICAM-1 and sL-selectin levels increased in all the three groups 180 min after ROSC. At baseline, sICAM-1 concentrations were higher in the ZDF group compared with the ZLC group (*p* < 0.05). No difference was seen at the end of the experiment. In the SprD group, sL-selectin was higher both at baseline (*p* < 0.05) and 180 min after ROSC (*p* < 0.05) compared with the ZDF group.

## Discussion

In the current study, we demonstrate that animals with T2DM compared with both groups of non-diabetic animals namely ZLC and SprD rats have more severe neurological injury following cardiac arrest, higher cytokine levels, and generalized metabolic derangement.

### Neurological injury

A major finding in the current study is the indication of increased neurological injury, observed in the ZDF group as higher levels of NSE. This observation may relate to the high glucose levels observed following cardiac arrest in the ZDF group. Hyperglycemia after cardiac arrest has been linked with poor neurological outcome in both experimental and human studies [18–21]; however, it is unclear whether this is caused by direct neurological injury, cardiovascular failure, or other pathologic processes. A direct effect on neurological injury is supported by a meta-analysis including animal models of the middle cerebral artery occlusion that showed increased cerebral infarct size in dextrose induced hyperglycemic animals [22]. In contrast, Nehme et al. [4] have in a retrospective study shown that mild pre-hospital hyperglycemia (8.0–11.9 mmol/L) independent of diabetes following out-of-hospital cardiac arrest may increase survival to hospital discharge. If diabetic animals were treated with glucose-lowering drugs, it might have been possible to avoid the detrimental effects of hyperglycemia. We refrained from administering glucose-lowering drugs to minimize differences between the groups, as most conventional glucose-lowering treatments have shown independent protective properties in the setting of IR-injury [23]. Elevated NSE levels are the major indicator of increased brain damage in the ZDF rats; however, little is known about NSE levels during the acute phase (within hours) of the post-cardiac arrest syndrome in humans [24]. Kang et al. [25] showed NSE levels of 2.9 μg/L and 4.5 μg/L after 1 and 6 h of reperfusion in non-diabetic rats following 5 min of cardiac arrest, which is similar to what we observed in the SprD group. Neurological function in the current study was only evaluated by NSE levels and visible generalized tonic-clonic muscle contractions, which may be considered as a potential limitation. Notably, studies have shown association between neuronal injury examined by histology, neuro-functional tests, and NSE levels [26–29].

### Cardiac function

The cardiac function parameters were ambiguous, and due to the low number of animals with echocardiographic parameters in the ZLC group, the data must be interpreted with caution. We saw no difference in the cardiac output between ZDF and SprD groups; TnT values were highest in the ZDF group although not significantly, whereas, fractional shortening suggested increased left ventricular function in the diabetic group at the end of the experiment. However, fractional shortening may be an inadequate measure of cardiac function in this study as the decreased LVID and concomitant increased IVS and LPW thickness in the ZDF group may potentially result in an increased fractional shortening even in the presence of an unaltered or perhaps lowered stroke volume. This is supported by our finding of no difference in the cardiac output after cardiac arrest in the ZDF and SprD groups. The decreased diastolic diameter and increased wall thicknesses in the ZDF group may reflect edema and ischemic contracture due to IR-injury. This is supported by higher levels of TnT in the ZDF group although not significant. While previous studies in chronic diabetic rats have shown increased susceptibility to IR-injury [12, 30], this depends among other aspects on the age of the included animals as studies in isolated perfused hearts have shown increased hemodynamic recovery in 12–16-week-old ZDF rats, when compared with non-diabetic controls [31, 32]. Considering this, the increased fractional shortening in the ZDF group could be explained by this as our rats were 16–18 weeks old. We chose this age span to ensure that the induction of cardiac arrest was conducted on animals with fully developed T2DM, which were still resilient enough to survive the systemic IR-injury.

### Inflammatory response

The inflammatory response to resuscitation after cardiac arrest is complex, with an early activation of endothelial cells, cytokine response, and upregulation of adhesion molecules [33]. A pro-inflammatory response has been seen in patients with T2DM outside the setting of cardiac arrest, as several studies have shown, e.g., increased levels of IL-6 in T2DM patients [34–36]. It is therefore reasonable to believe that T2DM could affect the post-cardiac arrest inflammatory response. Interestingly, we showed no difference in cytokine levels between the groups prior to cardiac arrest. However, the higher cytokine plasma levels in the ZDF group following cardiac arrest could be a manifestation of increased IR-injury, as especially high plasma levels of IL-6 following the immediate phase after ROSC have been associated with cardiovascular dysfunction and a lower 30-day survival in humans [37, 38]. The high IL-10 levels might be a result of a preserved balance between the pro- and anti-inflammatory cytokines, as IL-10 is considered as one of the major anti-inflammatory cytokines [39]. Whether the elevated cytokine levels were due to a direct effect of diabetes on the inflammatory response elicited by IR-injury or other pathological process needs further investigations. Greater sICAM-1 levels in the ZDF vs. ZLC group at baseline is consistent with the previous literature [14]. Surprisingly, we saw higher sL-selectin levels in the SprD group. The fact that this was true both before and after cardiac arrest points in the direction of a between race difference in selectin expression.

### CPP and ROSC rates

Although a significantly greater CPP was achieved during resuscitation in the ZDF group vs. the ZLC and SprD groups, the ROSC rate did only differ between the ZDF and ZLC group, being the lowest in the latter group. In humans, a CPP above 15 mmHg is associated with an increased chance of achieving ROSC [40]. In our study, the mean values in all groups were above this limit, but one animal in the ZLC group achieved a substantially higher CPP, which could mask a trend of the overall lower perfusion pressures in this group. The study by Paradis et al. also indicates a correlation between increased maximum CPP values above 15 mmHg and higher ROSC rates, which could explain the increased ROSC rate in the ZDF rats compared with ZLC rats. The mechanical chest compression device was adjusted within a narrow range for the compression depth to correspond to one third of the thorax height and do not give an apparent explanation for the differences in CPP. Whether the difference in ROSC rates is related to the presence of hyperglycemia during resuscitation or is caused by cardiovascular changes induced by T2DM remains speculative.

### Limitations

The study has a number of limitations. The surprisingly low number of animals in the ZLC group achieving ROSC limits our ability to detect the differences between the groups following ROSC. Although total asphyxia times were similar between the SprD and ZDF rats, the no-flow time was increased in the SprD group making direct comparability difficult. The ZDF and ZLC rats are from the same inbred race of rats, opposed to the outbred and more genetic heterogenic SprD rat. This complicates the between-group comparison and may inflict unknown confounders to our results. The relative short reperfusion duration of 3 h limits the results to apply only to the acute setting of the post-cardiac arrest syndrome, which lasts multiple days in the clinical setting. Pilot studies showed that a longer reperfusion period was impossible due to premature death in the ZDF group. The limited blood volume of the rats precluded repeated blood samples and limited the number of biochemical analyses why we focused on neurological and cardiovascular function, as they are the most common causes of death following cardiac arrest [41]*.* Our results suggest a potential impact of T2DM on the post-cardiac arrest syndrome, but further studies are needed to investigate the mechanism behind IR-injury in the setting of T2DM.

## Conclusions

In a cardiac arrest model, neuronal injury is increased in T2DM animals compared with non-diabetic controls. Although this study lacks to uncover the specific mechanisms causing increased neuronal injury, the establishment of a cardiac arrest model of T2DM lays the important foundation for further experimental investigations within this field.

## Abbreviations

BE: Base excess
CPP: Coronary perfusion pressure
FiO_2_: Fraction of O_2_ in inspiratory air
IR-injury: Ischemia-reperfusion injury
IVSD: Interventricular septum in diastole
IVSS: Interventricular septum in systole
LVIDD: Left ventricular inner diameter in diastole
LVIDS: Left ventricular inner diameter in systole
LVOT: Left ventricular outflow tract
LVPWD: Left ventricular posterior wall in diastole
LVPWS: Left ventricular posterior wall in systole
MAP: Mean arterial pressure
NSE: Neuron-specific enolase
PaCO_2_: CO_2_ partial pressure in arterial blood
PaO_2_: O_2_ partial pressure in arterial blood
ROSC: Return of spontaneous circulation
SD: Standard deviation
SprD: Sprague Dawley
T2DM: Type 2 diabetes mellitus
ZDF: Zucker diabetic fatty
ZLC: Zucker lean control
